# Management of Biliary Stricture Following Emergent Pancreaticoduodenectomy for Trauma: Report of Two Cases

**DOI:** 10.7759/cureus.2829

**Published:** 2018-06-18

**Authors:** Sharjeel Israr, Nathan S Rubalcava, Jordan A Weinberg, Michael Jones, Thomas L Gillespie

**Affiliations:** 1 General Surgery, St. Joseph Hospital and Medical Center, Phoenix, Arizona, Usa, Phoenix, USA; 2 Trauma Surgery, St. Joseph Hospital and Medical Center, Phoenix, Arizona, Usa, Phoenix, USA; 3 Trauma Surgery, Creighton University School of Medicine, Phoenix, USA

**Keywords:** pancreaticoduodenectomy, trauma whipple, biliary-enteric stricture

## Abstract

Stricturing of the biliary-enteric anastomosis is a known complication of emergent pancreaticoduodenectomy (PD) performed for trauma. Percutaneous techniques have become the first-line option for the management of these strictures. In cases where percutaneous intervention fails, surgical revision of the biliary enteric anastomosis is necessary. We present two cases of young males with penetrating injuries to the pancreatic head managed with PD and subsequently developed post-operative biliary strictures. The biliary stricture was managed successfully with percutaneous intervention for one of the patients. The other patient required surgical revision of the biliary anastomosis.

Pancreaticoduodenectomy is typically performed in patients with malignant or benign biliary obstruction with associated ductal dilatation. In the setting of trauma, the bile duct is typically non-dilated, creating greater susceptibility for anastomotic stricture. Although such strictures may be amenable to percutaneous cholangioplasty, strictures involving distal anastomoses may require operative revision. Thus, we suggest creating the more proximal hepaticojejunostomy during the initial operation, as this may benefit the success of percutaneous management should a stricture develop. Operative revision is the definitive management of post-PD biliary stricture.

## Introduction

Major pancreaticoduodenal injuries are seen uncommonly, but can be devastating and difficult to control when encountered. The incidence of injury after a traumatic abdominal insult, blunt or penetrating, is reported at <5% for the panceaticoduodenal complex [1]. Although rare, an emergent pancreaticoduodenectomy (PD) for trauma is recommended when the injury results in devitalization of the pancreatic head, disruption of the pancreaticoduodenal complex, or significant destruction of the second portion of the duodenum. Emergent PD for trauma, also known as trauma Whipple, has been used for decades in patients with no alternative management options and peri- and post-operative mortality rates for this procedure are reported between 16-60% [2-3]. Despite early recognition of injury and adequate operative intervention, the subsequent hospital course is often prolonged and fraught with complications.

Hemodynamic instability and metabolic derangements further complicate the technical difficulty of this procedure. Nevertheless, high-grade damage to the pancreaticoduodenal complex warrants resection and reconstruction. Consequently, diversion of biliary, pancreatic, and gastric flow is needed and may be performed concurrently, or staged. Anastomotic strictures are a common occurrence after PD for trauma; however, the incidence is not as well-reported as it is for elective PD for malignant or benign disease [4]. The incidence of biliary strictures may be higher in trauma Whipple patients than those getting a PD for malignant or benign disease due to size of ductal lumen; however, that has yet to be reported in the literature. When a patient presents with symptoms of obstructive jaundice and radiographic evidence of anastomotic stricture is found, management requires a multidisciplinary team approach. This includes trauma surgeons, interventional radiologists, and hepatobiliary specialists. In recent years, percutaneous techniques have become the first-line option for the management of these strictures, which have the benefit of avoiding the morbidity of operative re-exploration and more feasibility than endoscopic repair [4-5]. Nonetheless, in cases where percutaneous interventions fail, an operation to revise the biliary-enteral anastomosis may be necessary.

We retrospectively compared management approaches for biliary anastomotic strictures following PD for penetrating pancreatic trauma in two cases. Both cases were performed by a single surgeon at an academic level-one trauma center. A literature search resulted in no such case report. Presented below are two cases of young males with penetrating pancreaticoduodenal injuries managed with PD that subsequently developed post-operative biliary anastomotic strictures. One patient was managed successfully with percutaneous intervention and the other required surgical revision of the biliary-enteric anastomosis.

## Case presentation

Case 1: An 18-year-old male sustained a single gunshot wound to the abdomen. Exploratory laparotomy demonstrated the following injuries. Liver laceration was found in segment five, it was controlled with two sutures on a blunt needle and hemostatic matrix. The colon was found to have avulsion of the mesentery with obvious ischemia to the hepatic flexure, secondary to transection of the middle colic vessels. It was managed with right hemicolectomy and end ileostomy. Duodenum was found to have an entrance and exit wound involving the ampulla of Vater and distal common bile duct. He underwent PD with choledochojejunostomy, pancreaticojejunostomy, and gastrojejunostomy at the index operation.

**Figure 1:**
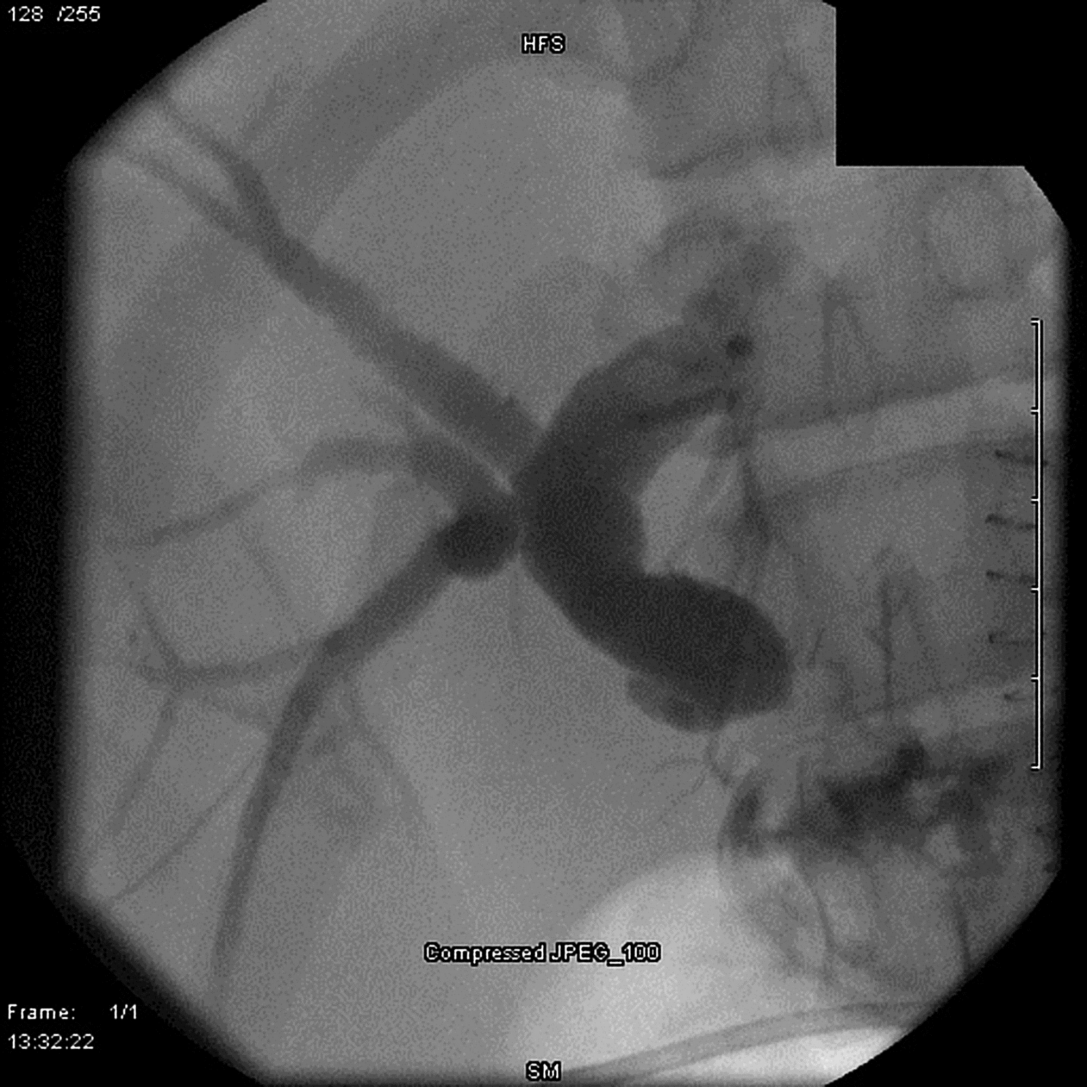
Biliary anastomotic stricture following choledochojejunostomy.

**Figure 2:**
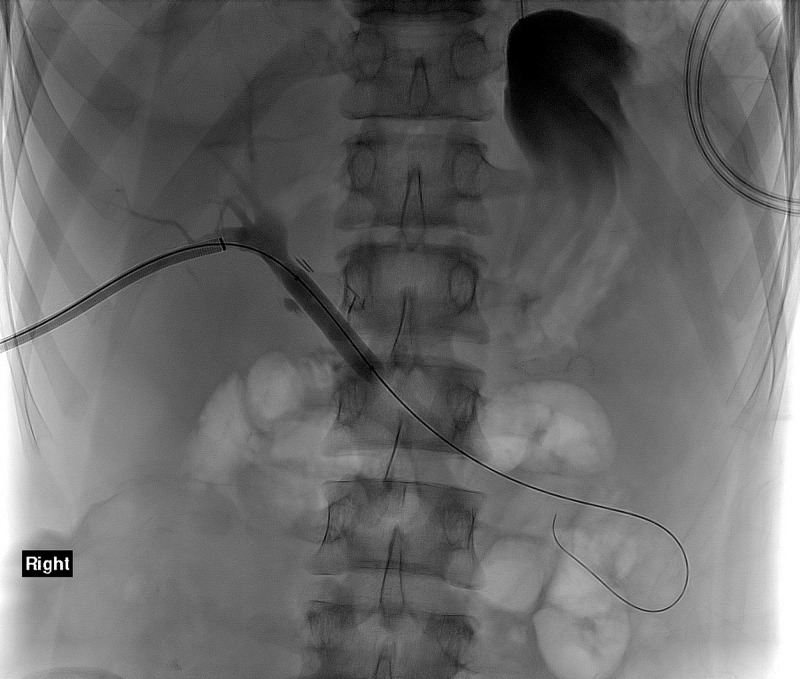
Balloon angioplasty of strictured choledochojejunostomy.

His post-operative course was complicated by a high-grade obstruction at the biliary anastomosis (Figure 1). Serial attempts at percutaneous cholangioplasty and stenting (Figure 2) were unsuccessful, which was demonstrated by persistently elevated total serum bilirubin levels (>2.0mg/dL). Therefore, long-term percutaneous transhepatic drainage (PTD) was performed. After several months of catheter decompression, a subsequent attempt at cholangioplasty was again unsuccessful. The patient then underwent operative revision of the anastomosis to hepaticojejunostomy. He had no procedural complications and no evidence of restricturing at three-month follow-up.

Case 2: A 16-year-old male sustained multiple gunshots and presented to the trauma bay in hemorrhagic shock. He was taken immediately for emergency laparotomy and injuries identified were injury to two segments of the small bowel, injury to the inferior vena cava (IVC), grade four injury to the right kidney, and destructive injury to the head of the pancreas. Right nephrectomy, IVC ligation, small bowel resection, and PD without reconstruction were performed at first operation. At planned re-exploration on postoperative day two, biliary continuity was accomplished with hepaticojejunostomy.

The patient had a complicated post-operative hospital stay, including ventilator-acquired pneumonia and hemodialysis requirement. However, he was discharged home in good health after 68 days. Three years later, the patient developed symptomatic stricture of the hepaticojejunostomy. ERCP was attempted but without success in reaching the biliary limb. The stricture was managed by interventional radiology using percutaneous transhepatic catheterization with serial balloon dilation. The transhepatic catheter was removed after six months, and the patient has no signs of obstructive jaundice six months later.

## Discussion

Emergent PD for penetrating trauma was first reported in 1961 by Howell et al. Since then, emergent PD for trauma has been only reported in small series. Operative mortality after blunt or penetrating trauma to the pancreas and duodenum have been reported between 15-60% [3,6-7]. The largest series of trauma Whipple (n=18) was reported by Krige et al. [3] in 2014 and demonstrated mortality of 15.8%. Nevertheless, when non-repairable injuries to the pancreaticoduodenal complex are found – including pancreas, duodenum, common bile duct, or ampulla of Vater – PD should be performed.

Long-term anastomotic stricture formation is frequently seen in PD; however, the incidence and management following trauma Whipple have not been well-reported, compared to Whipple procedure for malignancy. Biliary strictures in patients with malignant or benign disease is associated duct dilatation. In contrast, for cases of emergent PD performed for trauma, the patient will typically have a non-dilated bile duct that makes surgical anastomosis more technically demanding and also may increase susceptibility to anastomotic stricture formation. This could especially be true for young patients with much smaller ductal lumen size.

Hepaticojejunostomy anastomotic strictures are typically amenable to correction by percutaneous cholangioplasty or ERCP. However, strictures involving more distal anastomoses (i.e. choledochojejunostomy), as demonstrated above in the first case, may require operative revision if multiple attempts of less invasive symptomatic management remain ineffective. It is conceivable that fibrosis from initial damage and diminished blood supply may render percutaneous intervention less effective for stricture of choledochojejunostomy performed for trauma compared with hepaticojejunostomy.

## Conclusions

Stricturing of the biliary-enteric anastomosis is a known complication of emergent pancreaticoduodenectomy (PD) performed for trauma. The two cases presented herein are demonstrative of our experience with the clinical course of this complication. Percutaneous techniques have become the first-line option for the management of post PD biliary anastomotic strictures. When performing the biliary-enteric anastomosis, we advise creating a relatively proximal anastomosis (forming a hepaticojejunostomy) as this may benefit the success of percutaneous management should a stricture develop. Operative revision is the definitive management for biliary stricture following PD for trauma.
